# A Comprehensive Review on Corn Starch-Based Nanomaterials: Properties, Simulations, and Applications

**DOI:** 10.3390/polym12092161

**Published:** 2020-09-22

**Authors:** Chella Perumal Palanisamy, Bo Cui, Hongxia Zhang, Selvaraj Jayaraman, Gothandam Kodiveri Muthukaliannan

**Affiliations:** 1 State Key Laboratory of Biobased Material and Green Papermaking, College of Food Science and Engineering, Qilu University of Technology, Shandong Academy of Science, Jinan 250353, China; perumalbioinfo@gmail.com (C.P.P.); zhanghongxia326@hotmail.com (H.Z.); 2 Department of Biochemistry, Saveetha University, Chennai, Tamil Nadu 600077, India; jselvaendo@gmail.com; 3 Department of Biotechnology, School of Bio Sciences and Technology, Vellore Institute of Technology, Vellore, Tamil Nadu 632014, India; gothandam@gmail.com

**Keywords:** corn starch, nanomaterials, corn starch materials, instrumental methods, biomedical applications

## Abstract

Corn (*Zea mays* L.) is one of the major food crops, and it is considered to be a very distinctive plant, since it is able to produce a large amount of the natural polymer of starch through its capacity to utilize large amounts of sunlight. Corn starch is used in a wide range of products and applications. In recent years, the use of nanotechnology for applications in the food industry has become more apparent; it has been used for protecting against biological and chemical deterioration, increasing bioavailability, and enhancing physical properties, among other functions. However, the high cost of nanotechnology can make it difficult for its application on a commercial scale. As a biodegradable natural polymer, corn starch is a great alternative for the production of nanomaterials. Therefore, the search for alternative materials to be used in nanotechnology has been studied. This review has discussed in detail the properties, simulations, and wide range of applications of corn starch-based nanomaterials.

## 1. Introduction

*Zea mays* L. is a member of Poaceae family that is generally called corn and maize. It has been developed as a staple food in several region of the globe [1]. Hitchcock and Chase in 1971 explained the botanical features of corn [2]. A heavy, vertical, solid stem and large, thin leaves form a tall annual grass. The female inflorescences (pistillate) that develop to be the torn ears are spikes with a consolidated hub, bearing matched spikelets in longitudinal columns; each line of combined spikelets as a rule makes two lines of grain. The yellow and white corn assortments are the most famous as food, although red, blue, pink, and dark piece assortments are regularly grouped, spotted, or stripped. Every ear is enclosed by customized leaves called shucks or husks [3]. Mangelsdorf (1950) reported that the corn initially originated from America, and it was originally revealed by Christopher Columbus in 1492. It is one of the main food sources worldwide. In the 1600s and 1700s, the Americans used corn as their staple food, and in the 1800s, corn turned out to be one of the important commercial crops [4].

Corn is broadly categorized into six varieties—namely dent corn, flint corn, pod corn, popcorn, flour corn, and sweet corn. The assortment of corn-based human food incorporates grinding the corn into cornmeal or masa, squeezing into corn oil, and obtaining a mixed refreshment beverage such as whiskey bourbon as a result of fermentation, distillation, and chemical feedstock [5].

**Figure 1:**
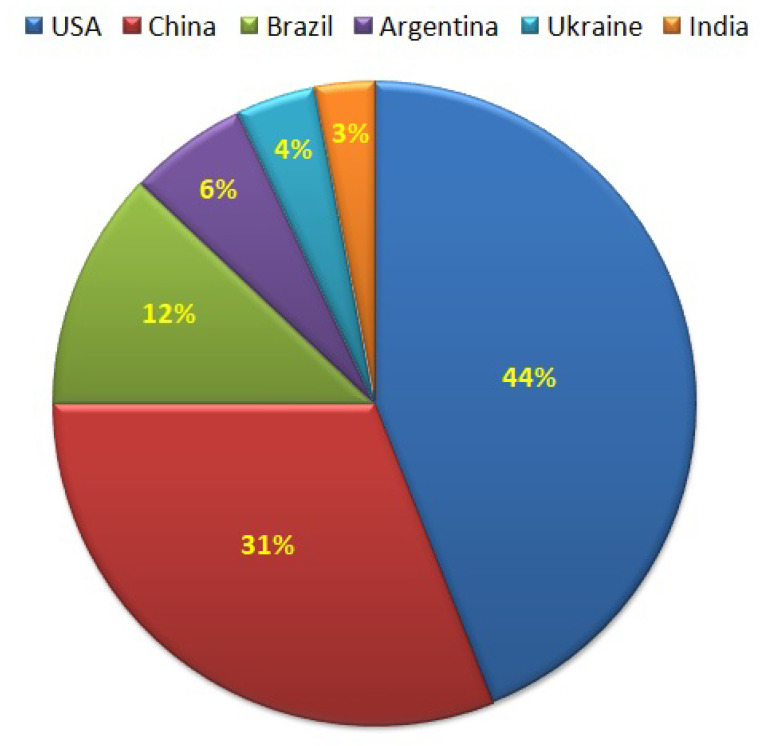
Corn production worldwide.

Corn is extensively cultivated all over the world, and a large quantity of corn is cultivated every year (Figure 1). As per the International Grains Council 2013, the total world production was 1.04 billion tons. In America, corn is one of the major grains; the country produced 361 million metric tons of corn in 2014. Over the past few years, corn farmers experienced a stable hike in yearly revenues. In 2016/17, the U.S. delivered more than 33% of the overall corn production.

In 2016, the United States traded almost 56.5 million metric tons of corn, making the country the world’s greatest corn exporter. Japan and Mexico were the most significant purchasers of U.S. corn in 2015, purchasing around 12.1 million metric tons and 11.31 million metric tons, respectively. Global corn creation measurements in 2019 clarified that the United States was the fundamental maker of corn creation; the amount adding up to about 366.3 million metric tons. China produced 257.3 million metric tons and Brazil produced 94.5 million metric tons, adjusting the top corn delivering nations (www.statista.com).

Zhao et al. (2008) declared that corns are rich in dietary fiber, nutritional supplements (vitamins A, B, E, and K), minerals (magnesium, potassium, and phosphorus), phenolic acids and flavonoids, plant sterols, and various phytochemicals (lignins and bound phytochemicals). However, the different assortments of corn have impressively various phytochemical profiles concerning flavonoids and carotenoids. Blue, red, and purple corn have a higher grouping of anthocyanidins (up to 325 mg/100 g DW corn) with cyanidin subsidiaries (75–90%), peonidin derivatives (15–20%), and pelargonidin subordinates (5–10%). Yellow corn is an excellent source of carotenoids (up to 823 g/100 g DW corn) with glutein (half), zeaxanthin (40%), β-cryptoxanthin (3%), β-carotene (4%), and α-carotene (2%). High-amylose corn is rich in amylase (up to 70%, all things considered) [6].

Next to corn grain, sweet corn is utilized as one of the most popular vegetables in North America and China, and its notoriety has expanded across the globe. Sweet corn is one of the top six vegetables utilized in the United States [7]. Canned and solidified sweet corn ranks third, placing it in the middle of vegetables utilized in the United States. [8]. This review discusses the potential of corn starch-based nanomaterial properties, simulations, and their wide range of applications.

## 2. Starch

**Figure 2:**
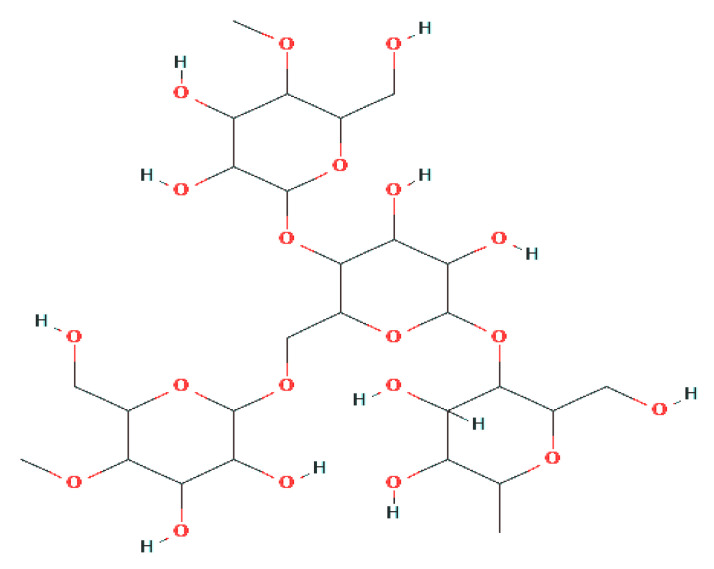
Basic structure of the starch molecule.

Starch is commonly known as amylum, and it is a polymeric carbohydrate consisting of a large number of glucose units connected by glycosidic bonds (Figure 2). It is recognized as a carbohydrate in human diets. It occurs in many staple foods such as potatoes, wheat, corn, rice, and cassava [9]. Pure starch extracted from plants was converted into flour-like white powder, which is insoluble in water [10]. This powder contains minute granules, and the width varies from 2 to 100 µm, and it has a thickness of about 1.5 µm. The fundamental formula of this polymer is (C_6_H_10_O_5_)n, and the glucose monomer is called α-D-glycopyranose (or α-D-glycose). In the view of their botanic source, starch crude materials contain different trade factors, sizes, shapes, and concoction content [11].

In many industries, starch is used as emulsifiers, viscosifiers, defoaming agents for encapsulation, and as sizing agents. In the detergent industries, starch is used in the production of biodegradable, non-toxic, and skin-friendly detergents. In many chemical industries, it is used for the production of surfactants, polyurethanes, resins, and in biodegradable plastics. It is also used in the construction industries for concrete admixtures, plasters, and insulation, as well as in oil drilling, mineral, and metal processing [12]. In food industries, starch is improved into sugars, for instance by malting, and the starch is fermented to form ethanol, which is used in the production of whiskey (by brewing) and biofuel. Inside the pharmaceutical business, starch was used as an excipient, pill crumble, and folio [13]. Corn also produces high amylose starch; it has an elevated level of gelatinization temperature compared to other types of starch and maintains its resistant starch content during baking, mild extrusion, and in further food processing techniques [14]. It was utilized as an insoluble dietary fiber in processed foods—for example, bread, pasta, cookies, crackers, pretzels, and other low moisture foods [15]. It has been suggested that starch gives the medical advantages of unblemished entire grains [16].

### Corn Starch

Around 80% of the world’s creation of starch is corn starch, which was extracted from corn pieces (content 64–80%) through the wet-processing process [17]. Corn starch is used in a broad variety of foodstuffs and applications. Basic corn starches have a small amount of protein (0.35%), lipid (0.8%), ash, and >98% of two polysaccharides, namely amylose and amylopectin. Starch comes from plant sources that are insoluble in water, and at room temperature, it is in the form of granules [18]. Usually, corn and waxy maize starch granules differ in their size from 2 to 30 mm; most fall in the range of 12–15 mm. They also differ in shape, appearing as cross-sections of polygons [19].

## 3. Preparation of Corn Starch Nanomaterials

Nanotechnology is one of the fastest-growing studies, which includes science, medical, engineering, and technology at the nanoscale level; mainly, this was used to form the nanoparticles ranging from 1 to 100 nm size [20]. In modern days, the use of nanotechnology in the food industry has developed as a defense against biological and chemical worsening; it has also improved the bioavailability, enrichment of physical properties, and other areas. However, it has limits in commercial-scale usage, since it has been expensive. Hence, there is a need to search for substitute materials that should be inexpensive to be utilized in the nanotechnology [21]. Starch is a biodegradable natural polymer for the production of nanocrystals or nanoparticles. These types of materials could be produced by a variety of techniques, using chemical, enzymatic, and physical treatment, and they may be used as a drug transporter, as a quality indicator for foodstuffs (nanoencapsulation), and in the reinforcement biodegradable and non-biodegradable polymeric matrices [22].

Silva et al. (2018) mentioned that acid hydrolysis is one of the best commonly used methods to make corn starch nanocrystals. This method contains two-step hydrolysis reactions: the first one is fast hydrolysis, and the second one is slow hydrolysis. Some researchers reported that there are three significant strides in acid hydrolysis: fast, slow, and moderate [23]. In the initial step, the hydrolysis of the indistinct pieces of the granules is attacked, as the moderate advance is the weakening of the crystalline districts [24]. Corn starch nanocrystals formed in this technique have high crystallinity and a platelet-like figure. At last, for a homogeneous dispersal of the nanocrystals, the suspension is subjected to a mechanical process [25].

### 3.1. Preparation of Corn Starch Nanomaterials with Natural Polymers

Corn starch has been genuinely and synthetically tweaked to improve its qualities in order to fit it for a couple of uses (Table 1). Modification was done to satisfy the prerequisite of precise properties [26]. Four types of technique were generally used to change the corn starch: physical, substance, enzymatic, and hereditarily alterations [27]. Copolymers have the capacity to create warm, physical, mechanical, and compound properties of corn starch. The aftereffect of regular and engineered polymers on the properties of corn starch has been broadly examined [28].

Fabra et al. (2016) made the thermoplastic corn starch (TPCS) nanobiocomposites, which have bacterial cellulose nanowhiskers (BCNW) [29]. Ghanbarzadeh et al. (2010) made new altered starch/carboxymethyl cellulose (CMC) composite films by an immediate strategy. The advancement of biodegradable materials dependent on starch has become an exceptionally alluring choice, and the creation of starch-based plastics are addressing significant worry related to the health of the planet [30].

Teaca et al. (2013) arranged and analyzed the impact of natural acid tweaked starch microparticles/plasticized with glycerol biocomposite films accomplished by the fuse of 10, 20 and 30 wt % birch cellulose (BC) inside a glycerol. A plasticized framework made with the corn starch (S) and concoction changed the starch into miniaturized scale particles (MS). The expansion of cellulose close to the modified starch miniaturized scale particles slightly improved the starch-based film’s water obstruction. Some retreating of water take-up for each predetermined time was observed for most of the tests containing 30% BC [31].

Mendes et al. (2016) built up the biodegradable polymer mixes from corn starch and thermoplastic chitosan using the expulsion technique. The result of this examination affirmed that it conceivably produced effective cornstarch–chitosan mixes by expulsion by a high scattering. This kind of mix has some potential applications in bundling, especially where an enormous amount of the prepared polymer is basic when contrasted with cluster handling [32]. Paiva et al. (2018) arranged the support of thermoplastic corn starch with cross-connected starch/chitosan microparticles. Microparticles of corn starch and chitosan cross-linked with glutaraldehyde and delivered by the dissolvable trade strategy are read as fortification fillers for thermoplastic corn starch plasticized with glycerol. The nearness of 10% w/w chitosan in the microparticles is demonstrated to be basic to ensuring successful cross-linking, as exhibited by water solvency [33].

Alves et al. (2015) analyzed the properties of corn starch/gelatin/cellulose nanocrystal (CNC) films. The results from this examination indicated that the convergence of gelatin and CNC lead to an increase in the film thickness, quality, and extension at break [35]. Rodríguez-Castellanos et al. (2015) created the gelatin–corn starch polymer lattice strengthened with 2,2,6,6-tetramethylpiperidine-1-oxyl (TEMPO)–cellulose utilizing twin-screw expulsion and pressure forming [36]. Chen et al. (2017) built up the combinations of gelatin (G) and oxidized corn starches (OCS) that were found as another microcapsule complex for single bead splash drying. The drying and breakdown attributes of composite beads have been determined with assistance of the single drop drying method [38].

Feltre et al. (2018) created the thermal-resistant corn starch alginate dots by dribbling agglomeration. This examination researched the agglomeration of local cornstarch and the creation of microcapsules by trickling sodium alginate suspensions into a calcium chloride arrangement. The cross-linking response shaped a calcium alginate that filled in as an epitome lattice and covered the cornstarch granules [23]. Cordoba et al., (2013) investigated the impact of starch filler on calcium–alginate hydrogels stacked with yerba mate cancer prevention agents. A fluid concentrate of yerba mate (Ilex paraguariensis) with antioxidant properties was typified in calcium–alginate hydrogels containing corn starch as filler at various concentrations. The addition of starch improved the exemplification proficiency from 55% to 65% [39]. Fujiwara et al. (2013) produced and portrayed the alginate–corn starch–chitosan microparticles containing stigmasterol through the outer ionic gelation procedure. The measure of stigmasterol in the oil recouped from microparticles was 9.97 mg/g. This method showed that the microencapsulation of stigmasterol is achievable [40].

Xiong et al. (2013) investigated the impact of a castor oil advancement layer created by response on the properties of polylactide/hexamethylenediisocyanate–starch mixes. Lv et al. (2015) examined the effect of strengthening at various temperatures on the thermal properties of poly (lactic corrosive)/starch mixes. Toughening was seen as supportive to debilitate and even take out the enthalpy unwinding close to the glass change temperature. Although the strengthening expanded the examples’ crystallinity, it affected the thermal steadiness of the PLA/starch mixes [41,43].

Soybean has particular dietary benefits due to its high protein, nutrients, and minerals that offer solid favorable circumstances. The sticking thickness of both ordinary and waxy corn starch with soy protein was surprisingly expanded by dry warmth treatment. Well-being and therapeutic advantages related with incorporated soy protein include diminished blood cholesterol level, insurance against cardiovascular malady, and a decreased danger of specific malignancies (prostate and breast) in people [55,56,57].

Ji et al. (2017) explored the rheological properties and microstructure portrayal of typical and waxy corn starch when dry warmed with soy protein detached. The outcomes demonstrated that the sticking consistency of both typical and waxy corn starch with soy protein was surprisingly expanded by dry warmth treatment [46].

Collagen is the fundamental portion of the connective tissue in vertebrates and has an orderly structure. It is answerable for 25–35% of absolute protein in body. Collagen films are generally more grounded than other protein films; however, it loses its unique quality in water bit by bit. So as to get by during troublesome handling conditions, various synthetic concoctions—for example, aldehydes—are utilized as cross-connecting specialists in collagen packaging arrangement with corn starch [58,59].

Wang et al. (2017) created and investigated the mechanical properties and solvency of corn starch–collagen composite films in water. Checking electron microscopy pictures uncovered that starch–collagen films had a more unpleasant surface contrasted with unadulterated collagen films, which became smoother after warming [48].

Fatty acids have been incorporated into biopolymer films in order to reduce their water vapor permeability, which is relatively high in polysaccharide-based films, such as corn starch, due to their highly hydrophilic nature [60]. Jimenez et al. (2010) contemplated the effect of re-crystallization on pliable, optical, and water fume hindrance properties of corn starch films containing unsaturated fats. Starch films containing glycerol were joined with unsaturated fats, so as to diminish the hygroscopic character of the films and to improve water fume penetrability [61].

Glycerol is a lackluster, scentless, thick fluid that contains three hydroxyl bunches propane-1,2,3-triol, and the consolidated corn starch with glycerol, which explain its hygroscopic character and dissolvability in water. The conventional applications of glycerol incorporate its consolidation into the food industry, the creation of pharmaceuticals, and individual consideration items, while it is the enemy of coolers, herbal concentrates, and numerous different procedures as a middle of the road compound [62,63].

Chen et al. (2017) examined the glycerol focus on corn starch morphologies and gelatinization practices amidst warm treatment. At the point when corn starch granules with no additional glycerol were treated at 65 °C, the granules of corn starch were totally broken and firmly associated, and the trademark birefringence of the starch granules vanished. Different minute procedures uncovered that the starch gelatinization was postponed to higher temperatures as the glycerol fixation expanded. Within the sight of glycerol–water frameworks (5%, 10%, 20%, and half, *w*/*w*), the pinnacle temperatures of corn starch expanded by 1.6, 7.4, 10.7, and 19.7 °C, individually, which contrasted with corn starch in water. The quick visco-analysis sticking profiles indicated that the gelatinization temperature expanded with the expansion of glycerol focus, which was steady with enraptured light magnifying instrument perceptions and DSC tests [50].

Poly-glutamic corrosive is a decomposable regular biopolymer. It is made out of nonstop units of “D-glutamic corrosive” and L-glutamic corrosive”. It is soluble in water. The corn starch with poly-glutamic acid is utilized for some applications such as in water treatment, beautifying agents, sedate conveyance, tissue designing, biological glue, and as an oil-decreasing operator, among others [64,65].

Xu et al. (2016) built up the starch–poly-glutamic acid join copolymers by microwave illumination. It is normal practice to join poly-glutamic acid in another polymer. One method of delivering join copolymers is warming in a microwave. A microwave is helpful in light of the fact that it gives a scope of warming levels and weight [52].

Algae are exciting natural resources, as they have a rapid growth rate and are available in different environments but do not hinder the food production. They can be used as an alternative source for plastic and bio-based packaging materials [66]. A large scale of microalgae can be investigated as a biodegradable matrix strengthener to achieve a number of advantages. Nannocloropsis gaditana, a microalgae with an adaptable cell membrane and hard cell wall, contains large amounts of polyunsaturated fatty acids, oils, and antioxidants, as well as pigments that provide packaging materials and edible coverings with functional properties [67,68].

Seaweeds have abundant polysaccharides that have been shown to be suited for biodegradable polymers. The most frequent polysaccharides derived from seaweed are alginates, agar, and carrageenan; they have been extensively investigated in various fields such as tissue engineering, pharmacology, food, and textiles. It is a kind of red kelp that is broadly developed for the creation of the hydrocolloid known as kappa carrageenan (κ-carrageenan) [69]. The combination of *K. alvarezii* seaweed and corn starch used for the production of films with or without built-in fillers with broad mechanical quality and other productive properties required in numerous modern applications [70,71]. Khalil et al. (2018) synthesized composite films with different *K. alvarezii* seaweed and corn starch concentrations. They found that the highest tensile strength and the greatest possible elongation at break were a composite film with 3% seaweed and 1% starch. With the inclusion of seaweed, both the mechanical characteristics and hardness of the composites have been enhanced. With an increase in the concentrations of starch and seaweed, the water vapor permeability of the composite films also increased linearly [54].

### 3.2. Preparation of Corn Starch Nanomaterials with Synthetic Polymers

The corn starch nanomaterials combined with synthetic polymers were described in Table 2. Polyvinyl alcohol (PVA) is acquired from monomers of vinyl. The hydrolysed polyvinyl Acetate is an engineered water dissolvable polymer. The materials used as manures, pesticides, herbicides and fungicides and to cover seeds in agribusiness is colorless and odorless. It was likewise used to make medical procedure strings, implants, scaffolds for cells culture and artificial organs [72].

Tian et al. (2017) developed the polyvinyl alcohol (PVA)/corn starch blend films with enhanced properties; the films were fabricated by melt processing and montmorillonite (MMT) reinforcing. The MMT nanolayers could act as heat and mass transport barriers and retard the thermal decomposition of the composites. It is the largest produced synthetic water soluble polymer that has valuable properties such as biodegradability, biocompatibility, chemical resistance, and good mechanical properties [73].

Polycaprolactone (PCL) is a straight, hydrophobic, and reasonably crystalline polyester of engineered biodegradable polymer that can be slowly used by organisms. It is a profoundly biocompatible aliphatic polyester that has a warm high atomic weight (40,000 and 80,000 g mol^−1^) PCL with high-amylose corn starch (HA-CS, 70% amylose) and waxy corn starch granules, just as the non-granular starch acetic acid derivation subsidiaries. The film properties of these mixtures were evaluated using differential calorimetric scanning, dynamic mechanical thermal analysis, and Instron tensile testing [74]. The PCL/HACS mixtures were the strongest with ≈15% lower tensile strength and a 50% higher modulus than PCL, and up to 25 wt % HA-CS. Optical microscopy indicated that the small size of the HA-CS granules (10 μm) and great scattering of the granules in the PCL grid were the explanations behind the contrasts in the great mechanical properties in the different mixes. Cross-linking was found to improve the warm properties of the mixes [75].

Khalil et al. (2014) developed nanocomposite blends utilizing thermomechanical preparation that was comprised of starch and polybutylene. The nanocomposite blends have been studied by integrating microscopic observational knowledge for each step in selective extraction. These experiments allowed the identification, until the full continuity of every phase, of blend compositions that are commensurate to the beginning of partly continuous percolation [77].

Corn starch combined with polyethylene has an excellent hydrophobic nature, and it also shows high viscosity, hydrogen bonding ability with ether oxygen, and biocompatibility. It has been widely utilized in the field of biomedicine due to these properties. It also recognizes applications in various industrial technologies, including cosmetics [84]. Ortega-Toro et al. (2016) studied the better properties of glycerol by mixing polycaprolactone and/or polyethylene glycol plasticized starch films in a low-level ratio. With 5% PCL, starch films lead to stabilizing and extending films. Although starch/PCL film is the key concern, it demonstrates the phase separation of incompatible polymers and poor interface adhesion, since the chemical interaction between polymers is lower [78]. Jagadish and Raj (2011) studied the properties and sorption of polyethylene oxide blended films. These films showed various thermal, barrier, mechanical, and optical characteristics along with surface morphology and sorption characteristics. The sorption data are very useful in order to properly select water–vapor barrier packaging materials for packaging. Poly(ethylene oxide) (PEO)/starch in PEO/starch combinations are noteworthy as they redetermine their use as a biological packaging medium [85].

Polyacrylic acid (PAA) consists of acrylic acid monomers. Every monomer contains a carboxylic group on it. It has a high negative charge. Its temperature of 106 °C is significant for the glass transition. Starch-based polymers with super sponges are modest and have amazing water maintenance qualities, which is the reason they are of extraordinary modern significance. They can retain and hold a huge measure of fluid. The polymers, which consist of homo and copolymers, have three-dimensional structures [86]. Bin-Dahman et al. (2015) studied the compatibility of PAA and maize starch mixes with different techniques. The blends were made with the help of glycerol as a plasticizer using the solution mixing and casting method. Studies of X-ray diffraction (XRD) showed that its crystalline structure was broken by the injection of PAA into starch. By varying the composition, the morphology of the mixes was changed. Scanning electron microscopic (SEM) analyses have shown that PAA was produced at greater starch loads in terms of layers around starch granules [79]. Dang et al. (2017) characterized the PAA grain starch (PACS) blend as a chemical sand fixing substance. Mixing PA with CS was used to make the PACS mix. The inter-molecular interactions between the components of the mixture were found by FTIR. The continuous mixture phase with good sand fixation capacity was confirmed by SEM analysis [87].

Polystyrene is a thermoplastic polymer that is commonly used for its outstanding mechanical qualities, biologic inertness, and low-cost inertia; it is highly efficient for packaging, consumer product, construction, medical applications, and production [88]. Gutiérrez and Alvarez (2017) reported a reactive extrusion into the two-screw extruder using zinc octanoate (Zn(Oct)_2_) as a catalyst, followed by the compressive molding of the native and oxidized corn (corn) starch/polystyrene (PS) blends, using glycerol as a plasticizer. Their study showed that the catalyst caused cross-linking between starch and PS and increased its reactivity by the oxidative modification of the starch. This rise in starch-modified structures hydrophobicity was attributed to the addition of carboxyl and carbonyl groups into the structure of starch [80]. Men et al. (2015) prepared a copolymer of polystyrene-grafted starch-g-PS with a large graft percentage by the application of methylimidazolium acetate ionic liquid 1-ethyl-3 as a solvent and potassium persulfate as an initiator. Their research has shown that prior to polystyrene grafting, ionic liquid starch dissolution is a multiple synthesis methodology for amphiphylic graft copolymers based on polysaccharide, which has a high percentage of graft [89].

Polyethylene glycol (PEG) is an unbiased polyether that is water solvent, non-poisonous, and has low reactivity. An amphilipic atom containing hydrophilic and hydrophobic moieties, regardless of whether straight or fanned, terminated with hydroxylic groups and a general [HO-(CH_2_CH_2_O)n CH_2_CH_2_-OH] structure. The concentration of PEG-iso intercoms affects the melting and glass transition temperatures, storage, and loss modules of high amylose starch (HAGS)-PEG-PU films significantly [90]. Tai et al. (2017) developed starch–polyurethane (PU) composite films with improved mechanical and hydrophobic properties [81].

Ethylene–vinyl acetate copolymer (EVA) is a commercial polymer utilized in a few applications such as polymer compatibilization and as a medium of dispersion of low-energy polymer fillers. One interesting aspect of EVA is the fact that more polar copolymers can be easily modified [91]. Da Róz et al. (2012) prepared strong mixes of hydrolyzed ethylene vinyl copolymers and thermoplastic maize starch. The findings revealed that TPCS’s breakage, thermal, and water absorption characteristics have been improved by the inclusion of EVA in TPCS for young modules, tensile power, and elongation. These compatible mixtures are good for replacing polymers based on petroleum due to their low cost and biodegradability [82].

Polyvinyl fluoride (PVDF) is a semi-crystalline, thermoplastic polymer with mechanical and chemical resistance. Future applications as piezoelectrical, pyroelectrical, and ferro-electrical materials are of great concern to PVDF, in particular with respect to sensors and actuators [92]. Azevedo et al. (2014) reported mixtures of PVDF or poly (viN-trifluoroethylene) P(VDF-TrFE) compounded by maize starch and obtained a biocompatible material [47].

### 3.3. Preparation of Corn Starch Nanomaterials with Organic Materials

The efficient carrier for bioactive chemicals, plant extracts, and nutrients is corn starch-dependent nanomaterials [93]. It is a new solution for oral administration to prevent early inactivation for safety reasons, as certain conditions limit the bioavailability of active ingredients or medications [94]. The prepared maize starch-based nanomaterial with diameters of 1 to 100 nm enables increased levels of organic materials in cells and tissues and also enhances the shelf life by slowing down deliveries [95]. Despite such advantages in terms of antioxidants, free radicals, and antitumors, among others, the industry has stepped up work in this area to maximize the health benefits obtained from nutraceutical formulations [96]. Li et al. (2016) prepared the premix membrane emulsion (PME) uniform starch microcapsules for avermectine (Av) under controlled release. The PME process was used for preparing high production levels of starch microcapsules. Kinetic analysis revealed that non-Fickian and Case II transportation were involved in Av release provisions. In order to achieve satisfactory release profiles, the diameter (0.70–4.8 μm) and Av contents (16–47%) were changed [97].

Farrag et al. (2018) developed nanoparticles of starch loaded with quercetin by the process of nanoprecipitation. In vitro studies in the preparation of nanoparticles loaded with quercetin were performed at 35% ethanol as a release medium. The origin of starch affects the percentage of quercetin load, and the kinetic release and antioxidant activity of the nanoparticles are generated. The cereal nanoparticles of starch and quercetin displayed the smallest amount and the lowest proportion of quercetin release relative to tuber and legume nanoparticles. Fickian diffusion appears to be mainly controlled by the release kinetics that were revealed that fit the release data to the Peppas–Sahlin model [98].

### 3.4. Preparation of Corn Starch Nanomaterials with Inorganic Materials

The new class of nanocomposite materials on the boundary between material science, life science, and nanotechnology is bionanocomposites. They may be defined as a combination of an inorganic or organic solid, which has at least one dimension in the nanoscale range of the biological degradable polymer (such as starch) [99]. When the solid mode has biomineralization and no corrosion effects, the bionanocomposites can also be considered as a “green bionanocomposite” and are environmentally safe [100]. Research studies aimed at the production of entirely biodegradable bionanocomposites also extract knowledge from synthetic nanocomposites dependent on polymer. Several types of nanofillers have also been integrated in starch matrices to improve their physical characteristics [101].

Moreira et al. (2013) investigated a sequence of bionanocomposite films using Mg(OH)_2_ based on corn starch, which provided the mechanical strengthening of the nano-sized brucite. Wet precipitation was synthesized and incorporated into matrices of starch at different concentrations (0–8.5% by weight) by brucite nanoplates with an aspect ratio of 9:25. In the starch bionanocompounds, SEM observed a high degree of nanoplate dispersion and strong interfacial adhesion between the filler and the matrix. TGA revealed an association between starch and brucite that changed its breakdown profiles [102]. Prusty and Swain (2016) prepared thin films with various nano CaCO_3_ compositions in an aqueous medium starch hybrid nanocomposite polyethylhexylacrylate (PEHA)/polyvinyl alcohol (PVA) [103]. Yao et al. (2011) prepared biodegradable hybrid films with the sol–gel approach to the starch/polyvinyl (PVA)/nanosilicone (nano-SiO_2_) alcohol. Due to the application of nano-SiO_2_, the crystal structure of the films was improved. Nano-SiO_2_ is also applied to prolong the film aging. However, the enzymatic degradation test shows that added nano-SiO_2_ has no substantial impact on film biodegradability [104].

## 4. Morphological and Physiochemical Characterization

### 4.1. Atomic Force Microscopy

Nanoparticles are becoming increasingly important in many areas, including catalysis, biomedical applications, and information storage. These materials are superior because of their unique size-dependent properties. The atomic force microscope (AFM) allows simulation and analysis on a three-dimensional basis; individual particles and particle classes may be solved as compared to other microscopic techniques [105]. AFM is one of the most popular forms of microscopy scanning samples. From an experimental point of view, nanoparticles measure AFM tip modifications or nanoparticles manipulation; the interaction of nanoparticles with the AFM probe has been considered quite extensively. When spherical nanoparticles are placed on an ideally flat substratum, they can be easily determined by the measurement of a nanoparticle height from the AFM image [106]. This quantity is not influenced by the effects of a tip-sample convolution and can yield accurate tests for nanoparticles. Consequently, nanoparticles’ statistical results rely on the proper choice and correct use of AFM data assessment algorithms that add a human error to the whole process of measurement [107].

### 4.2. Transmission Electron Microscopy

Transmission electron microscopy (TEM) technology is used to picture a nanoparticle sample using an electron beam, providing a much higher resolution than light-based imaging techniques [108]. TEM is the best way to precisely calculate the thickness, grain composition, size, and morphology of nanoparticles. TEM has also recently been used as a means of determining nanocomposite effects on biological systems [109].

### 4.3. Scanning Electron Microscopy

One of the common methods used to imagine the microstructure and morphology of a material is through scanning electron microscopy (SEM). For material characterization (including biomaterials), different modes of SEM are available: X-ray mapping, the secondary imaging of electrons, backscattered imaging electrons and electron channels, and the electron microscopy of Auger. For the analysis and interpretation of micron or nanometer-scale observations, SEM can be used [110]. The resolution of an electron microscope for field scanning can be as low as 1 nm. Another key element of SEM is it enables a three-dimensional observation and analysis of samples due to its deep field depth. The greater the depth of field, the more sample information that is provided [111].

### 4.4. Universal Tensile Machine

The nanofibrous mats were measured with a Universal Tensile Machine (UTM) with tensile or mechanical power. The traction test tests the force needed to split a specimen and how far the specimen stretches to that point. The stress and load movement diagram are generated by a tensile check, which is used for tensile modulus determination [112].

### 4.5. Fourier Transform Infrared Spectroscopy

By analyzing the vibrant frequencies of the chemical bonds, Fourier transform infrared (FTIR) spectroscopy provides useful insight into the functional groups in the structure. The vibratory activation intensity of the molecules is between 10^13^ and 10^14^ Hz, which is infrared radiation. This allows IR spectroscopy to analyze and conduct quantitative and qualitative studies of self-assembled functional groups organized in the nanoparagon surfaces [113]. FTIR enables interfaces to be analyzed in situ to investigate the functional group surface adsorption on nanoparticles. The advantage of FTIR is that it allows users to analyze the layer and the overlapping phase of nanoparticles covered on the ATR element. The molecular data collected by this technique help users determine the structural and conformational modifications of the self-assembled functional coordinating groups on nanoparticle surfaces [114].

### 4.6. Thermal Methods

Highly advanced techniques are available for the characterization of morphology and structure of polymers such as differential scanning calorimetry (DSC), thermogravimetric analysis (TGA), thermomechanical analysis (TMA), and dynamic mechanical analysis (DMA). The material can often be identified and quantified based on its characteristic thermal stability and transition temperature and by investigating the changes in the measured property (e.g., enthalpy, weight, length, stiffness, etc.) with temperature [115].

### 4.7. Differential Scanning Calorimetry

Differential scanning calorimetry (DSC) is a thermal analysis technique that calculates the energy absorbed or emitted by a sample as a function of temperature. A DSC equipment diagram, enthalpy, entropy, and special heat determination can be used to detect the sample’s temperature and the amount of heat flow [116].

### 4.8. Thermogravimetric Analysis

Thermogravimetric analysis (TGA) is a thermal analysis method in which the mass of a sample is measured over time as the temperature changes. It is used to measure the thermal stability of a sample. This analysis includes knowledge about physical phenomena such as phase transition, ingestion, absorptions, adsorption, and desorption, as well as chemical phenomena such as chemisorptions, thermal decomposition, and nano-solid gas reactions. The temperature or time curve is also called as the thermogravimetral curve, the data with respect to the change in the mass with temperature/time of the sample using TGA is shown as a graph/curve. A derivative plot of the TGA curve, called DTG, shows the rate at which mass changes and shows the rate of mass loss versus temperature curve. Sample mass changes may occur because of processes such as evaporation, dryness, desorption or adsorption, sublimation, and thermal decomposition. These mass shifts are indicated as phase changes in the TGA curve or DTG curve peaks [117].

### 4.9. Differential Thermal Analysis

Differential thermal analysis (DTA) is a technique similar to that of differential calorimetry scanning thermoanalytics. In DTA, the substance under review is subjected to undergo thermal cycles, (i.e., the same of cooling or heating), so any variations in the samples during the analysis are recorded, and their comparisons are reported. DTA curves give data on changes such as glass transitions, crystallization, melting, and sublimation [118].

### 4.10. Dynamic Mechanical Analysis

Dynamic mechanical analysis (DMA) is a method in which the kinetic properties of the sample are studied by calculating the strain or stress that is generated as a result of strain or stress, which varies with the time (oscillating) applied to the sample. DMA is used for measuring polymer materials of various types using different deformation modes. Tension, compression, dual bending of cantiles, three-point bending, and shear modes exist, and according to the sample shape, modulus, and measuring purpose, the best type should be selected [119].

### 4.11. X-Ray Diffraction

X-ray diffraction (XRD) is an effective nanomaterial research tool (materials with structural properties in the range between 1 and 100 nm with at least one size). X-rays are a form of electromagnetic radiations, and the wavelength is radioactive. XRD is the main tool to test the nanomaterial structure. Quantitative, accurate information on the nuclear structures at interfaces can be provided by the intensities measured with XRD [120]. Nanomaterials have a proper length of the microstructure compared to the physical phenomena’s critical length scales, which give them specific mechanical, optical, and electronic properties. The XRD of nanomaterials provides a wealth of information, from phase composition to crystallite size, and from lattice strain to crystallographic orientation [109].

### 4.12. Nuclear Magnetic Resonance Spectroscopy Analysis

Nuclear magnetic resonance (NMR) is a powerful non-destructive analytical tool based on nuclei excitation by magnetic field exposure. It is a physical phenomenon in which a weak magnetic field (in the near field and thus without electromagnetic waves) disturbs the nuclei in a strong steady magnetic field and responds by generating an electromagnetic pulse in the magnetic field of the nucleus [121]. It is an electrical phenomenon in which NMR results from certain atomic nuclei’s special magnetic characteristics. NMR is also used regularly in sophisticated imaging methods, including magnetic resonance imaging (MRI). By measuring the return of the nuclei to their base level of energy, it provides detailed information on the molecular structure, dynamics, response status, and chemical environment [122].

### 4.13. Dynamic Light Scattering

Dynamic light scattering (DLS) is a technique in physics that is used for determining the size distribution of small particles in suspension and/or polymers in a solution. For DLS, the amplitude or photon self-correlation method (also known as photon correlation spectroscopy or almost-elastic light scattering) is typically used to analyze temporal variations. DLS is also used to test fluids, including condensed polymer solutions [123,124].

### 4.14. Rheological Analysis

Rheological analysis is an examination that is routinely measured by a rheometer that measures the flow of a fluid and the deformation of materials under applied strengths. Rheological properties assessment extends for all products ranging from oils such as polymers or surfactants’ dilutive solutions to condensed protein blends, pastes, creams, liquid, or solid polymers and asphalt [125]. The properties are measured with a mechanical rheometer or on a micro-scale by a viscometer or optical technique, such as the microrheology, from bulk sample deformation. It can be used for identifying the physical properties of a nanoparticle, with size as a sensitive tool [126].

### 4.15. Zeta Potential Analysis

Zeta potential analysis is a technique that is used to determine the surface load of nanoparticles in solution (colloids). The surface load of the nanoparticles attracts a thin layer of oppositely charged ions that are present on the nanoparticles [127]. This double film of ions diffuses into the solution along with the nanoparticles. The electrical potential at the border of the dual layer is known as the particles’ zeta potential and has values that typically range between +100 mV and −100 mV. The magnitude of the zeta potential for colloidal stability is predictive. Zeta nanoparticles that have values greater than +25 mV or less than −25 mV usually have a high degree of stability. Dispersions with a low potential value eventually aggregate due to Van Der Waals inter-particle attraction. Zeta potential is an important tool to understand the surface state and long-term stability of the nanoparticles [128].

## 5. Biomedical Application of Corn Starch Based Nanomaterials

**Figure 3:**
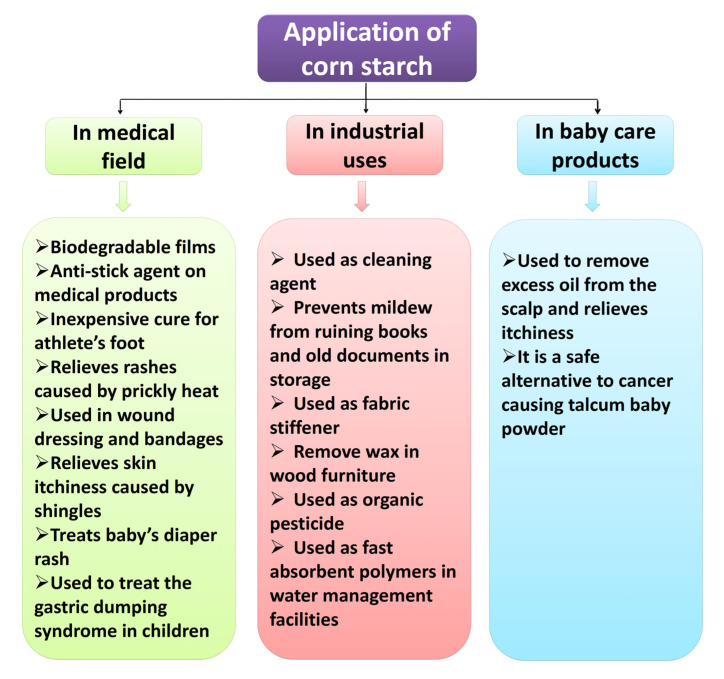
Different applications of corn starch.

The unique physiochemical and functional characteristics of natural starches such as their good biocompatibility, biodegradability, non-toxicity, and degradation make them useful for a wide range of biomedical applications (Figure 3). Several biodegradable starch polymers, particularly in the field of bone tissue technique, drug delivery systems, and hydrogels, have been broadly examined during the last few years [129].

Salgado et al. (2005) have shown that the biodegradable bone cements, which are based on starch, can provide a temporary structural base and gradually vanish thereafter. Furthermore, biodegradable polymers based on starch have been reported for bone tissue engineering scaffolding [130]. According to Gomes et al. (2002), an ideal scaffold must be designed on the basis of a biomaterial that has adequate rates of degradation compatible with new tissue formation. Therefore, the choice of starch can be used for scaffolding applications [131]. In drug delivery systems, the further potential attraction of biodegradable polymers based on starch has been reported. The drug administration device of biodegradable starch is effective in deliverability without surgery [132]. The findings of the above studies show that the starch can be used in the medical industry as biomaterials. However, current biodegradable polymers based on starch clearly have lower mechanical characteristics, thus limiting the ability to be utilized in various biomedical applications. Therefore, the production of nanocomposite starch content was initiated to remove biodegradable starch limitations alone [133]. Liu et al. (2016) developed a nanocarrier-based starch nanoparticles in which four polyphenols are inserted: (+) -catechin, (−) -epicatechin, (−) -epigallocatechin-3-gallate, and proanthocyanidin. In addition, the methyl thiazolyl tetrazolium assay demonstrated low cytotoxicity and good biocompatibility [134].

Nanoparticles have been an important topic of research in the area of anti-cancer drug delivery. The distribution application of nanoparticles made of biodegradable materials such as polylactic acid, proteins, and polysaccharides has been documented. Polysaccharide systems are becoming increasingly important among all the studies because of their low toxicity, large abundance, and high biocompatibility. However, relatively, very few nanoparticle supply structures dependent on starch are recorded [135]. Continuation of the cell-, tissue-, or disease-specific release of therapeutic nanoparticles is a potentially powerful technology. Xiao et al. (2012) reported a drug known to maintain 5-fluorouracil (5-Fu) antitumor loading and release of the new drug carriers/dialdehyde starch nanoparticles (DASNP). The aldehyde group that significantly enhanced breast cancer cell inhibition (MCF-7) was conjugating 5-Fu, the model medicine, into nanoparticles [136].

## 6. Future Prospective and Conclusions

The natural polymer of corn starch (CS) is broadly accessible and can be effectively changed by various physical or chemical methods. Plastic waste is one of the main environmental threats, and it is minimized by biopols. They are used in various areas for valuable applications and can replace polymers based on petroleum. The combining of starch with natural polymers, synthetic polymers, and organic and inorganic nanoparticles is based on different chemistries and physical methods. Such CS combinations demonstrate ideal biocompatibility, physical characteristics, and degradation rates. The development of improved CS-based bionanocomposite film with increased elasticity, break resistance, more humidity adsorption capability, low water vapor permeability performance, greater tensile strength, and strong physical and mechanical properties in comparison to simple starch-based films is among the most technical advancements in the polymer industry. The films produced can potentially be used in the food and pharmaceutical industries as packaging material. In bone tissue, repairing the bone and neural tissue, and coating UV-sensitive materials, CS mixes, with other polymers (natural/synthetic) and inorganic nanoparts can be used. Therefore, the prospect of modified CS-based polymers is inciting to develop high-value goods in diverse fields for new applications.

## Figures and Tables

**Table 1:** Combination of corn starch with natural polymers, method of preparation, characterization techniques, and their applications.

S. No	Corn Starch Combination with	Method of Preparation	Size of the Particles	Characterization Techniques	Applications	References
1.	Cellulose	Nanobiocomposite: Electrospun method	100 μm	Scanning electron microscopy, Polarized light microscopy, Tensile properties, Water vapor permeability, Oxygen permeability, Contact angle measurements, Optical properties	Food packaging	[29]
		Biocomposite films	80 μm	Water vapor permeability, Moisture absorption, Solubility in water, Tensile properties, Thermal properties	Industrial relevance	[30]
		Biocomposite films	100 μm	X-ray diffraction, Fourier transform infrared spectroscopy, Scanning electron microscopy, Thermal analysis, Mechanical properties, Water uptake	Packaging applications	[31]
2.	Chitosan	Biodegradable polymer blends: Extrusion	50 μm	Fourier transform infrared Spectroscopy, X-ray diffraction, Scanning electron microscopy, Thermogravimetric measurements, Film thickness, Mechanical properties	Production of packaging materials	[32]
		Crosslinked microparticles	20 μm	Fourier Transform Infrared Spectroscopy, X-ray diffraction, Scanning electron microscopy, Total soluble matter, Thermogravimetric measurements, Tensile tests, Water absorption	Packaging materials	[33]
		Biocomposite films	10–20 μm	Attenuated total reflectance–Fourier transform infrared analysis, Scanning electron microscopy analysis, X-ray diffraction method, Physicochemical properties, Mechanical properties, Measurement of crystallinity, Moisture absorption measurements, Water vapor permeability	Food and pharmaceutical packaging applications	[34]
3.	Gelatin	Nanobiocomposite	10 μm	Scanning electron microscopy, Differential scanning calorimetry, Thermogravimetric measurements, Water vapor permeability, Film thickness measurements–digital micrometer, Film opacity, Mechanical properties	Food and pharmaceutical applications	[35]
		Polymer matrix: Twin-screw extrusion and compression molding	20–50 μm	Tensile mechanical tests, Residual moisture analysis, Scanning electron microscopy, Thermogravimetric analysis	Food and pharmaceutical applications	[36]
		Biocomposite films	50 μm	X-ray diffraction method, Fourier transform infrared spectroscopy analysis, Scanning electron microscopy analysis, Optical properties, Mechanical properties, Water vapor permeability, Oxygen permeability, Thermogravimetric analysis, Differential scanning calorimetry	Applications in edible food packaging	[37]
		Microcapsule composite: Glass-filament single droplet dying method	50 μm	Fourier transform infrared spectroscopy analysis, Scanning electron microscopy analysis, Gel time determination, Transparency determination, Viscosity determination, Drying and dissolution of the composite particle	Food and pharmaceutical applications	[38]
4.	Alginate	Agglomerated beads by dripping method	100 μm	Differential scanning calorimetry, Fourier transform infrared spectroscopy, X-ray diffraction, Scanning electron microscopy, Particle size measurements, Particle shape parameters, Particle density	Biomedical applications: Control the structure and function of the engineered tissue	[23]
		Hydrogel beads: Peristaltic pump	100 μm	Scanning electronic microscopy analysis, X-ray diffraction, Fourier transform infrared spectrometry, Humidity content measurement, Values of water activity, Bulk density analysis, Differential scanning calorimetry	Protect and deliver yerba mate antioxidants into food products	[39]
		Microparticles: External ionic gelation technique	10–40 μm	Average degree of deacetylation, Scanning electronic microscopy analysis, X-ray diffraction, Fourier transform infrared spectrometry, Intrinsic viscosity	Pharmaceutical applications	[40]
5.	Polylactic acid	Nanocomposite Blends	50–200 μm	Scanning electronic microscopy analysis, Fourier transform infrared spectrometry, Differential scanning calorimetry, Polarized optical microscopy, Mechanical properties measurements, Rheological characterization	Applications in packaging, biomedical, and agriculture fields	[41]
		Nanocomposite microfibers: Melt electrospinning method	200–500 μm	Scanning electronic microscopy analysis, FTIR sample techniques: attenuated total reflection, UV–visible spectrophotometer, Differential scanning calorimetry, Water contact angle measurements	Biomedical applications	[42]
		Nanocomposite blends: Extrusion molding	100 μm	Differential scanning calorimetric analysis, X-ray diffraction, Thermogravimetric analysis	Biomedical applications	[43]
		Bionanocomposite: Extrusion method	100 μm	Scanning electronic microscopy analysis, Thermal degradation, Enzymatic degradation test, Burial test	Applications in packaging, biomedical, and agriculture field	[44]
6.	Proteins	Bionanocomposite: Extrusion method	10–100 μm	Specific mechanical energy, X-ray microtomography, Bulk density, Piece density, Expansion ratio, Water absorption, Water solubility indices, Gel permeation chromatography, Texture analysis	Health and medicinal applications	[45]
		Cooled pastes	20 μm	Confocal laser scanning microscopy, Fourier transform infrared spectroscopy, rapid visco analysis, Steady flow properties, Amplitude sweep tests	Enhancing the quality of starch-based food products including buttermilk or salad dressings	[46]
		Biodegradable film blends: Extrusion	10–00 μm	X-ray diffraction, Scanning electron microscopy, Thermogravimetric analysis, Differential scanning calorimetry, Mechanical properties, Water vapor permeability, Optical properties	Innovation for application as a packaging material	[47]
7.	Collagen	Biodegradable film	20–50 μm	Optical properties, Scanning electron microscope, Mechanical properties, Film solubility in water, Differential scanning calorimetry, X-ray diffraction, Fourier transform infrared spectroscopy	Applications in bioengineering and biomedicine fields	[48]
8.	Fatty acid	Biodegradable film	10–60 μm	Film equilibration and storage, Moisture content, Tensile properties, X-ray diffraction, Water vapor permeability, Scanning electron microscopy, Atomic force microscopy, Optical properties	Application as a packaging material	[49]
9.	Glycerol	Biodegradable paste	20–50 μm	Confocal laser scanning microscopy, Scanning electron microscopy, Differential scanning calorimetry, rapid visco analysis	Used as a thickener, gelling agent, bulking agent, and water retention agent	[50]
		Bionanocomposite: Reinforcing method	5–100 μm	X-ray scattering, Scanning electron microscopy, Mechanical properties, Thermal analysis, Water uptake	Biomedical applications	[51]
10.	Poly glutamic acid	Graft copolymer	-NA-	Water absorption index, Carbon, hydrogen, and nitrogen analysis, Graft content, Graft efficiency, Graft frequencies, Rheological characterizations	Applications in drug delivery, food, water treatment, cosmetics, and other fields	[52]
11.	Microalgae	Biodegradable film	20–100 μm	Optical microscopy, Scanning electron microscopy, Water vapor permeability, Oxygen permeability, Contact angle measurements, Mechanical properties, UV-blocking capacity, Small and wide angle X-ray scattering	Biomedical applications	[53]
12.	Seaweeds	Biodegradable film	50–100 μm	Optical properties, Film thickness, Mechanical properties, Water vapor barrier property, Fourier transform infrared spectroscopy	Biomedical applications	[54]

**Table 2:** Combination of corn starch with synthetic polymers, method of preparation, characterization techniques, and their applications.

S. No	Corn Starch Combination with	Method of Preparation	Size of the Particles	Characterization Techniques	Applications	References
1.	Polyvinyl alcohol	Nanocomposites	100 μm	Fourier transform infrared spectroscopy, X-ray diffractograms, Transmission electron microscopy, Differential scanning calorimetry, Kinetics of water absorption, Thermogravimetric analysis	Applications in green packaging materials and biodegradable plastics	[73]
2.	Polycaprolactone	Biodegradable composite	10–100 μm	Optical microscopy Thermal analysis, Mechanical properties, Tensile properties.	Applications in packaging materials	[76]
3.	Polybutylene	Nanocomposite blends	75–320 μm	Scanning electron microscopy, Confocal laser scanning microscopy, Oxygen and water permeation, Water vapor sorption, Fluorescein desorption experiments	Application in active antimicrobial packaging put in direct contact with intermediate to high moisture foods	[77]
4.	Polyethalene	Biodegradable Film	20–60 μm	Atomic force microscopy, Scanning electron Microscopy, X-ray diffractograms, Fourier transform infrared spectroscopy in total attenuated reflection, Thermal properties, Physicochemical properties	Applications in packaging materials	[78]
5.	Polyacrylic acid	Nanocomposite blends	5–50 μm	Fourier transform infrared spectroscopy, Dynamic mechanical analysis, Differential scanning calorimetry, Thermal gravimetric analysis, Water uptake experiments, X-ray diffraction, Scanning electron microscopy	Applications in textiles, paints, papers, adhesives, water treatment, pharmaceuticals and others	[79]
6.	Polystyrene	Nanocomposite blends	20–100 μm	Fourier transform infrared spectroscopy, Differential scanning calorimetry, Thermal gravimetric analysis, Water uptake experiments, X-ray diffraction, Scanning electron microscopy	Applications in pharmaceuticals and food industries.	[80]
7.	Polyurethane	Composite film	50–100 μm	Acquiring microstructure images, Thermal properties and crystallinity, Dynamic mechanical properties, Wide angle X-ray diffraction, Light and polarized light microscopy, Contact angle	Applications in packaging materials	[81]
8.	Vinyl acetate	Nanocomposite blends	20–40 μm	Fourier transform infrared (FTIR) spectroscopy, Water uptake, Tensile tests, Dynamic mechanical analysis, X-ray diffraction, Scanning electron microscopy, Thermogravimetric analysis	Applications in green packaging materials and biodegradable plastics	[82]
9.	Poly(vinylidene fluoride)	Nanocomposite blends	10–800 μm	Fourier transform infrared (FTIR) spectroscopy, Tensile tests, Dynamic mechanical analysis, X-ray diffraction, Micro-Raman spectroscopy Scanning electron microscopy, Thermogravimetric analysis	Applications in drug delivery, food, water treatment, cosmetics and other fields	[83]
